# Stillbirth in pregnancies with flat OGTT: Placental clues to occult maternal dysmetabolism

**DOI:** 10.1002/ijgo.70683

**Published:** 2025-11-21

**Authors:** Emma Bertucci, Alessandra Danieli, Francesco Ricciardiello, Isabella Neri, Francesca Monari, Alessandro D. Genazzani, Gaetano Bulfamante, Antonio La Marca, Laura Botticelli, Fabio Facchinetti, Laura Avagliano

**Affiliations:** ^1^ Prenatal Medicine Center, Obstetrics and Gynecology Unit, Department of Medical and Surgical Sciences for Mother, Child and Adult University of Modena and Reggio Emilia Modena Italy; ^2^ Obstetrics and Gynecology Unit, Department of Medical and Surgical Sciences for Mother, Child and Adult University of Modena and Reggio Emilia Modena Italy; ^3^ Department of Biomedical, Surgical and Dental Sciences University of Milan Milan Italy; ^4^ Toma Advanced Biomedical Assays S.p.A Varese Italy; ^5^ Department of Pathology University of Modena and Reggio Emilia Modena Italy; ^6^ University of Modena and Reggio Emilia Modena Italy; ^7^ University of Milan Milan Italy

**Keywords:** flat oral glucose tolerance test, intrauterine fetal death, placenta, pregnancy outcomes, villous immaturity

## 1 INTRODUCTION

Stillbirth is one of the most devastating pregnancy outcomes and maternal glycemic dysmetabolism may contribute significantly to its occurrence.^1^ However, identifying glycemic dysregulation in pregnancy remains challenging and is usually based on fasting plasma glucose (FPG) and/or an elevated glucose response at 1 and 2 hours (h) during the 75‐g oral glucose tolerance test (OGTT). Gestational diabetes mellitus (GDM) is diagnosed when plasma glucose is ≥92 mg/dL fasting, ≥180 mg/dL at 1 h, or ≥153 mg/dL at 2 h.^2^ Although values below these thresholds are considered normal, a minimal glycemic rise after glucose intake—referred to as a flat OGTT—represents an atypical trend with uncertain significance. Different definitions have been proposed including a glucose rise of less than 6%, 16.5%, or 20% at 1 and 2 h^3^, ^4^, ^5^ depending on the criteria adopted by the authors. Additional definitions include a difference of less than 20–45 mg/dL between fasting and post‐load values,^6^ a difference of less than 30 mg/dL between peak and fasting glucose concentrations,^7^ or post‐load values remaining below 100 mg/dL.^8^ Variations in the definition of flat OGTT may influence the reported prevalence, which ranges from 3.9% to 35% of pregnancies.^3^, ^7^ Herein, we report 10 cases of stillbirth in women with flat OGTT. Although none met the diagnostic criteria for GDM, all showed placental abnormalities suggestive of glycemic dysmetabolism.

## 2 MATERIALS AND METHODS

### 2.1 Study design

Among singleton pregnancies undergoing 75‐g OGTT, we included stillborn cases with flat OGTT defined by FPG <92 mg/dL and post‐load glucose values increasing by <20% of the fasting level. Stillbirth was defined as fetal death occurring ≥22 weeks of gestation according to the WHO definition.

### 2.2 Placental analysis

Histopathologic evaluation of the placenta was performed as previously reported^9^, ^10^ in agreement with the Amsterdam consensus of international criteria.^11^ Briefly, placental and umbilical cord samples were collected by local pathologists in accordance with national guidelines, which prescribe systematic sampling of the maternal and fetal surfaces, membranes, and three umbilical cord segments (placental and fetal insertions, and mid‐portion). For each case, at least three full‐thickness sections of normal placental parenchyma and additional samples from gross lesions were collected for histopathologic assessment. All tissue specimens were formalin‐fixed, paraffin‐embedded, and processed for conventional histologic examination. Hematoxylin and eosin‐stained 4 μm sections were retrospectively reviewed by two authors, blinded to all clinical data. The reviewing authors were not involved in the original sampling procedures.

Delayed villous maturation was defined as a monotonous villous population (≥10 villi) with centrally located capillaries and reduced vasculosyncytial membranes, resembling early gestational villous morphology, and diagnosed when present in at least 30% of a full‐thickness placental parenchymal slide.^11^ Chorangiosis was defined by the presence of more than 10 capillaries per terminal villi, in at least 10 villi, in several regions of the placenta.^12^ Villous edema was defined in presence of accumulation of interstitial fluid, located between the capillaries within the villi and the trophoblastic covering of the villi, involving more than 15% of all distal villi.^13^

The present study received IRB approval (35265–November 24, 2021). The method of the study agrees with the Declaration of Helsinki.

## 3 RESULTS

**FIGURE 1:**
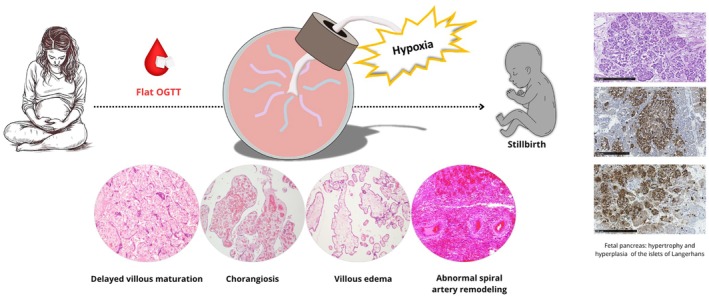
Flat oral glucose tolerance test (OGTT) and its association with placental maldevelopment and stillbirth. “Flat” OGTT indicates fasting plasma glycemia <92 mg/dL and post‐load glucose values increasing by <20% of the fasting level. For each case, at least three full‐thickness samples of the placental disc were analyzed. Placental samples were formalin‐fixed, paraffin‐embedded and processed for conventional histopathologic examination by light microscopy. Placental slides of 4 μm thick tissue sections, stained with hematoxylin & eosin, were jointly reviewed by two authors, blinded to all clinical information with the exception of gestational age. Histopathologic placental pictures show: villous immaturity with monotonous villous population observed in more than 10 of the same kind of villi, constituting at least one‐third of all the villi studied and indicating delayed villous maturation (staining: Hematoxylin–eosin; magnification: 20×); hypervascularity with extremely high number of capillaries in high number of villi scattered around several areas of the placenta, indicating chorangiosis (staining: Hematoxylin–eosin; magnification: 40×); accumulation of fluid in the villous interstitium, with disruption of the normal intravillous cytoarchitecture, indicating villous edema (staining: Hematoxylin–eosin; magnification: 40×); decidual arteriopathy with muscularized spiral arteries indicating abnormal spiral artery remodeling (staining: Hematoxylin–eosin; magnification: 20×). When available (*n* = 4), 4 μm thickness pancreas sections, stained with hematoxylin and eosin (scale bar 250 um) and immunostained with insulin antibody (IR002, Dako, Glostrup, Denmark), were examined by light microscopy, observing increased islets size related to both beta‐cell hyperplasia and hypertrophy (scale bar 250 μm).

Table 1 summarizes the case series. None of the women met the diagnostic criteria for glycemic dysmetabolism. However, placental histopathology revealed abnormalities commonly found in diabetic pregnancies: villous immaturity was observed in all cases. As shown in Figure 1, this lesion is characterized by fewer terminal villi than expected for the gestational age and by the presence of a monotonous villous population with thickened basement membrane, and centrally placed capillaries, resembling the histologic findings observed in early pregnancy, therefore is called “delayed villous maturation.” In this condition, the barrier for maternal‐fetal interchange of nutrients and oxygen is thickened, therefore delayed villous maturation could cause chronic fetal hypoxemia increasing the risk of perinatal mortality. The real mechanism underlying stillbirth in these cases is not fully understood but it is hypothesized that impaired placental maturation can reduce fetal resilience to stressors such as transient hypoxia from umbilical cord compression, as observed in five of our 10 cases.

**TABLE 1:** Clinical and histologic characteristics of 10 cases of stillbirth in pregnant women with flat OGTT.

Case No.	GA at Stillbirth (weeks) (index pregnancy)	Maternal age (years)	Maternal pre‐pregnancy BMI (kg/m^2^)	Other relevant maternal and/or pregnancy information	GA at OGTT (weeks)	Fasting plasma glucose value (mg/dL)	Glycemia: Value 2 (1 h) (mg/dL)	Glycemia: Value 3 (2 h) (mg/dL)	Fetal sex and birth weight (grams)	Stillbirth cause	Relevant placental and/or fetal histologic findings	Next pregnancy outcome
1	29	32	22.9	Asthma Atopic dermatitis	25	86	95	95	F 1340	Clinically unexplained—placenta abnormalities	Delayed villous maturation. Villitis of unknown etiology. Abnormal spiral artery remodeling. Fetal pancreas unavailable.	Gestational diabetes mellitus
2	29	38	24.8	‐	15	87	92	95	F 1330	Clinically unexplained—placenta abnormalities	Delayed villous maturation. Chorangiosis. Villous edema. Hyperplasia and hypertrophy of the fetal pancreatic islets of Langerhans.	No next pregnancy
3	31	32	27.1	‐	25	78	78	81	M 1300	Cord accident	Delayed villous maturation. Chorangiosis. Fetal vascular malperfusion. Velamentous cord insertion. Fetal pancreas unavailable.	Uneventful pregnancy though strict diet and lifestyle control due to preconceptional hyperinsulinism (Homa index 3.27)
4	32	38	24.9	Inherited thrombophilia (heterozygous mutation of factor V Leiden) found thanks investigation after previous deep vein thrombosis while taking EP pill	1st: 15 2nd: 24	86 78	97 86	89 91	F 1925	Clinically unexplained—placenta abnormalities	Delayed villous maturation. Acute atherosis of the maternal spiral arteries. Hyperplasia and hypertrophy of the fetal pancreatic islets of Langerhans.	No next pregnancy
5	34	33	22.4	Previous H‐SIL treated by LEEP	1st: 16 2nd: 26	84 71	74 95	82 84	M 1670	Clinically unexplained—Placenta abnormalities	Delayed villous maturation. Significant placental infarct. Fetal pancreas unavailable.	No next pregnancy
6	38.1	33	31.2	Polyhydramnios PCOS Hyperinsulinism	26	89	98	80	F 3000	Clinically unexplained—Placenta abnormalities	Delayed villous maturation. Chorangiosis. Massive fibrin deposition. Maternal floor infarct. Abnormal spiral artery remodeling. Hyperplasia and hypertrophy of the fetal pancreatic islets of Langerhans.	Two early miscarriages after the index pregnancy. Obstetric antiphospholipid syndrome at laboratory investigation. Fourth conceive after pre‐pregnancy 10% weight decrease; pregnancy uneventful with strict diet, lifestyle control and treatment with low dose aspirin and LMWH
7	38.4	38	25.7	Polyhydramnios	21	77	70	85	F 3500	Cord accident	Delayed villous maturation. Chorangiosis. True umbilical knot. Hyperplasia and hypertrophy of the fetal pancreatic islets of Langerhans.	Gestational diabetes mellitus
8	38.5	38	30	Autoimmune thyroiditis Polyhydramnios	24	81	87	97	M 2600	Cord accident	Delayed villous maturation. Fetal vascular malperfusion. Fetal pancreas unavailable.	No next pregnancy
9	39	32	22.2	Polyhydramnios	24	77	87	91	M 3300	Cord accident	Delayed villous maturation. Chorangiosis. Villous edema. Fetal vascular malperfusion. Fetal pancreas unavailable.	Gestational diabetes mellitus
10	39	37	24.1	PCOS Hyperinsulinism Autoimmune thyroiditis	24	91	100	101	M 4160	Cord accident	Delayed villous maturation. Chorangiosis. Fetal vascular malperfusion. Fetal pancreas unavailable.	No next pregnancy

*Note*: BMI, calculated as weight in kilograms divided by the square of height in meters. Histopathologic evaluation of the placenta was performed in agreement with the Amsterdam consensus of international criteria.^11^

Abbreviations: BMI, body mass index; EP, estro‐progestin; F, female; GA, gestational age; H‐SIL, high‐grade squamous intraepithelial lesion; LEEP, loop electrosurgical excision procedure; LMWH, low molecular weight heparin; M, male; OGTT, oral glucose tolerance test; PCOS, polycystic ovary syndrome.

Villous chorangiosis was present in six out of 10 cases. Figure 1 illustrates the typical features of this lesion characterized by an abnormally high number of capillaries (≥10) in more than 10 terminal villi. This finding reflects pathologic hypercapillarization with an excessive number of vessels within placental villi, likely resulting from an imbalance in angiogenic factors related to dysregulation of insulin and its receptors. Chorangiosis is associated with chronic hypoxia and/or hyperglycemia‐hyperinsulinemia, both of which can increase the risk of poor fetal outcomes, as occurred in our stillborn cases (Figure 1).

## 4 DISCUSSION

Our findings highlight 10 cases of stillbirth in women with flat OGTT, in which placental pathology revealed features indicative of glycemic dysmetabolism despite normal diagnostic testing for GDM. We acknowledge that the placental histopathologic abnormalities identified‐villous immaturity, delayed villous maturation, chorangiosis, villous edema, abnormal spiral artery remodeling—are not specific markers of diabetes mellitus rather being non‐specific findings that may occur in various other conditions.^14^ However, delayed villous maturation and chorangiosis are frequently associated with maternal diabetes^15^, ^16^ and once those placental and clinical findings (i.e., polyhydramnios, maternal obesity, PCOS, etc.) are considered simultaneously they may suggest undiagnosed or subclinical glycemic dysregulation capable of reducing fetal tolerance to hypoxic stress hence contributing to stillbirth. Moreover, it is worth noting that in our study five of the included women conceived again, and three of them developed overt GDM in subsequent pregnancies, reinforcing the likelihood of subclinical glycemic dysmetabolism during the index pregnancy.

The clinical implications of flat OGTT have been scarcely investigated and available results are conflicting. Some recent studies did not observe an increased risk of adverse fetal outcomes in pregnancy with flat OGTT,^3^, ^7^, ^17^ whereas others reported higher rate of complications, including fetal growth restriction, small for gestational age newborn and low Apgar score.^5^, ^8^, ^18^, ^19^ These discrepancies may reflect differences in the criteria used to define a flat OGTT, differences in sample size and variations in the ethnic composition of the study populations. Given these uncertainties, no specific changes in clinical practice are currently recommended, and neither capillary glucose testing nor continuous glucose monitoring is currently advised in this context. Future studies are needed because the mechanisms remain unclear, both regarding adverse fetal outcomes and the origin of the flat glycemic response. Glucose, the main fetal energy source, crosses the placenta through a concentration gradient with the fetus acting as a glucose sink (fetoplacental glucose steal phenomenon). During OGTT, enhanced fetal uptake may lower maternal glycemia, potentially explaining normal maternal results despite fetal signs of diabetic fetopathy, such as pancreatic islet hypertrophy/hyperplasia, observed in all four of our tissue‐available cases. In this context, a flat OGTT may reflect maternal glycemic dysregulation undetected by standard testing. An exaggerated fetoplacental glucose steal, compounded by fetal hyperinsulinemia, could distort maternal results and mask subtle maternal–fetal imbalance. Overall, a flat OGTT curve may not indicate normal glucose metabolism but rather suggest hidden dysregulation.

### 4.1 Strengths and limitations

The present study is strengthened by a systematic, standardized pathologic assessment according to international criteria, and by confirmation of a dysmetabolic milieu through fetal pancreatic histology and subsequent pregnancy evaluations, reinforcing the suspicion of subclinical maternal glycemic dysmetabolism. However, it is important to note that our data was unable to establish a causal relationship between maternal glycemic dysmetabolism and stillbirth. In some cases, fetal demise was attributable to other causes, including cord accidents, while in others, maternal comorbidities such as autoimmune disorders, thrombophilia, or obesity may have influenced placental findings, representing potential confounders. Additionally, the sample size was limited, reflecting the rarity of stillbirths within this specific population, which may affect generalizability. These observations gain further context when considering the multifactorial pathogenesis of fetal death, which results from the interplay of maternal, fetoplacental, and external stressor factors.^20^ Within this framework, impaired placental function due to the histologic lesions may act in concert with maternal and fetal factors (e.g., obesity, thrombophilia, autoimmune disease, polyhydramnios), lowering the threshold for adverse outcomes in response to transient stressors such as brief hypoxic episodes from cord compression.

Clinically, these findings highlight the potential contribution of subtle maternal metabolic alterations, even below GDM diagnostic thresholds, to placental dysfunction and adverse perinatal outcomes, with implications for risk stratification and monitoring in future pregnancies. In clinical practice, they guided our counseling on healthy lifestyle, including diet and physical activity, even in the absence of previously overt GDM but in the presence of a flat OGTT, with the aim of potentially improving outcomes in subsequent pregnancies.

## 5 CONCLUSION

In conclusion, to the best of our knowledge, this is the first report to describe an association between flat OGTT and stillbirth; herein we report a case series of stillborn in non‐diabetic pregnant women with flat OGTT curves. Although these women did not meet the criteria for GDM, the placental and fetal findings raise the possibility that flat OGTT may indicate subtle glucose dysregulation in pregnancy. Awareness of this pattern may help clarify its clinical significance and support closer clinical monitoring, to reduce the risk of fetal complications.

## AUTHOR CONTRIBUTIONS

EB, LA, GB and ALM conceived the original idea for this brief communication. FR, AD and FS conducted the literature searches, helped to identify previous work and describe the case report. FR, AD and FS wrote the first draft of the paper. GB and LA assessed the anatomic‐pathologic aspects. EB and LA made substantial and final revisions to the manuscript. All authors were involved in interpreting the findings and revising drafts and agreeing the final version.

## FUNDING INFORMATION

The present study did not receive any specific grants from funding agencies in the public, commercial, or not‐for‐profit sectors.

## CONFLICT OF INTEREST STATEMENT

All authors have no conflicts of interest to disclose.

## Data Availability

Data sharing is not applicable to this article as no new data were created or analyzed in this study.
